# Anatomy and Physiology of Neurons in Layer 9 of the Chicken Optic Tectum

**DOI:** 10.3389/fncir.2019.00063

**Published:** 2019-10-14

**Authors:** Marinus Kloos, Stefan Weigel, Harald Luksch

**Affiliations:** ^1^Department of Animal Sciences, Chair of Zoology, Technical University of Munich, Freising, Germany; ^2^Institute of Neuroscience, Technical University of Munich, Munich, Germany

**Keywords:** subcortical pathways, visual processing, circuit analysis, patch recordings, multimodal integration

## Abstract

Visual information in birds is to great extent processed in the optic tectum (TeO), a prominent laminated midbrain structure. Retinal input enters the TeO in its superficial layers, while output is limited to intermediate and deeper layers. In addition to visual information, the TeO receives multimodal input from the auditory and somatosensory pathway. The TeO gives rise to a major ascending tectofugal projection where neurons of tectal layer 13 project to the thalamic nucleus rotundus, which then projects to the entopallium. A second tectofugal projection system, called the accessory pathway, has however not been studied as thoroughly. Again, cells of tectal layer 13 form an ascending projection that targets a nucleus known as either the caudal part of the nucleus dorsolateralis posterior of the thalamus (DLPc) or nucleus uveaformis (Uva). This nucleus is known for multimodal integration and receives additional input from the lateral pontine nucleus (PL), which in turn receives projections from layer 8–15 of the TeO. Here, we studied a particular cell type afferent to the PL that consists of radially oriented neurons in layer 9. We characterized these neurons with respect to their anatomy, their retinal input, and the modulation of retinal input by local circuits. We found that comparable to other radial neurons in the tectum, cells of layer 9 have columnar dendritic fields and reach up to layer 2. Sholl analysis demonstrated that dendritic arborization concentrates on retinorecipient layers 2 and 4, with additional arborization in layers 9 and 10. All neurons recorded in layer 9 received retinal input *via* glutamatergic synapses. We analyzed the influence of modulatory circuits of the TeO by application of antagonists to γ-aminobutyric acid (GABA) and acetylcholine (ACh). Our data show that the neurons of layer 9 are integrated in a network under strong GABAergic inhibition, which is controlled by local cholinergic activation. Output to the PL and to the accessory tectofugal pathway thus appears to be under strict control of local tectal networks, the relevance of which for multimodal integration is discussed.

## Introduction

The vertebrate midbrain has recently come back into the focus of network analysis due to its central role for visual and multimodal processing (Basso and May, 2017; Ahmadlou et al., 2018; De Franceschi and Solomon, 2018; Herman et al., 2018; Beltramo and Scanziani, 2019). While the genetic amenability has also put the zebra fish model into center stage (Helmbrecht et al., 2018; Marachlian et al., 2018), the optic tectum (TeO) of birds has already been studied for a long time in a variety of species, thus adding a dimension of comparative analysis (Luksch, 2003; Wylie et al., 2009). The avian optic tectum offers several advantages: its distinct laminated architecture with 15 layers (Cajal, 1911) combined with its large size in relation to the entire brain facilitates network analysis and makes it a great model for the vertebrate midbrain in general. In contrast to its homolog in mammals, the superior colliculus (SC), visual input in the superficial layers 2–7 and output from the deeper layers 9–15 are spatially separated (Hunt and Webster, 1975; Hellmann and Güntürkün, 2001). The visual input is organized in a retinotopic manner, projecting a spatiotopic map on the tectal surface (Hunt and Webster, 1975). However, input is not limited to the visual modality, but auditory input has been shown to exist as well (Knudsen, 1982; Lewald and Dörrscheidt, 1998; Niederleitner et al., 2017), which has led to the optic tectum being considered a center of multimodal integration.

**Figure 1:**
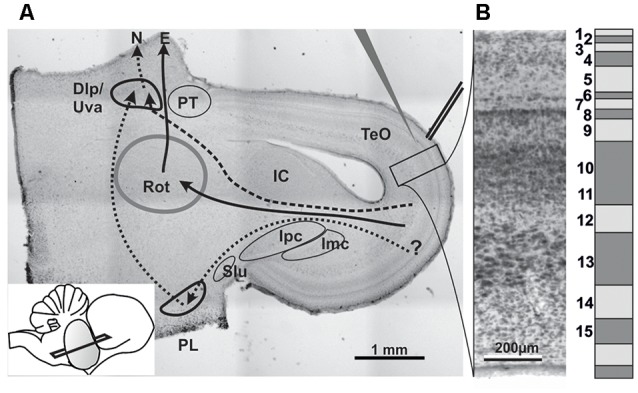
**(A)** Histological section stained with neutral red to illustrate the slice orientation used for patch experiments and the position of stimulation and patch electrodes. Major nuclei and projection systems are depicted. Dlp/Uva, Nucleus dorsolateralis posterior/Nucleus uvaeformis; E, Entopallium; IC, Inferior colliculus (=Nucleus mesencephalicus lateralis pars dorsalis); Imc, Nucleus isthmi magnocellularis; Ipc, Nucleus isthmi parvocellularis; N, Nidopallium; PL, lateral pontine nucleus; PT, Nucleus praetectalis; Rot, Nucleus rotundus; Slu, Nucleus isthmi semilunaris; TeO, Tectum opticum; solid arrows: tectofugal pathway; dashed arrows: accessory tectofugal pathway. Inset: slicing angle seen from lateral. Note that the outlines of the structures are idealized as some would not show up on that section plane. **(B)** Histological (left) and schematic depiction of the tectal layers in a chicken brain.

From the optic tectum, several ascending projections originate, which are collectively called the tectofugal pathway (Figure 1). This projection mainly consists of a very well characterized pathway from the retina to the nucleus rotundus (Benowitz and Karten, 1976; Luksch et al., 1998), *via* cells in layer 13 of the TeO, here called the major tectofugal pathway. However, a second, less well characterized tectofugal projection connects the retina to various thalamic and brainstem targets *via* cells in layers 8–15 of the TeO. This accessory tectofugal projection reaches a thalamic target which is termed the caudal part of the nucleus dorsolateralis posterior (DLPc) in the pigeon (Gamlin and Cohen, 1986) and the nucleus uveaformis (Uva) in the zebrafinch (Wild, 1994). Interestingly, this structure was originally described as a song control nucleus (Nottebohm et al., 1982). While direct tectal projections to the DLPc/Uva originate mostly in layer 13, there is an additional indirect projection that arises from several layers in the TeO and targets the lateral pontine nucleus (PL), which then projects upon the DLPc/Uva (Wild and Gaede, 2016). The PL also projects heavily to the cerebellum in pigeons (Clarke, 1977) and was shown to receive auditory information in cats (Aitkin and Boyd, 1978), and may thus provide the multimodal input to the DLPc/Uva shown in several studies (Korzeniewska and Güntürkün, 1990; Wild, 1994; Wild and Gaede, 2016). As the accessory tectofugal pathway thus seems to be involved in a complex network dedicated to multimodal integration, the question arises on which level of the pathway multimodal integration occurs.

Audio-visual integration has been well documented in the optic tectum of specialists such as the barn owl (Knudsen, 1982) but also behaviorally in other species, including generalists such as the chicken (Verhaal and Luksch, 2016a). In both species, the external nucleus of the inferior colliculus projects to the optic tectum (Niederleitner et al., 2017). The somatosensory aspect of multimodal integration, however, has not been addressed as frequently and remains largely unknown. Due to its proximity to the auditory system and clear evidence of somatosensory integration in the cat SC (Meredith et al., 1992; Stein and Meredith, 1993; Wallace et al., 1998), trimodal integration in the avian tectum appears to be very likely. Recently, the connection between the nucleus geniculatus lateralis pars ventralis and the nucleus intercalatus thalami has also been suggested as a possible structure involved in visual-somatosensory integration (Vega-Zuniga et al., 2018).

The processing of sensory information in the TeO is subject to an abundance of modulatory factors. In majority, these modulations are mediated by the neurotransmitters γ-aminobutyric acid (GABA) and acetylcholine (ACh). GABAergic inhibition has been identified to originate from horizontal cells in superficial layers and other GABAergic cells throughout the tectum (Tömböl and Németh, 1999; Luksch and Golz, 2003; Weigel and Luksch, 2012), but also in connections with the isthmic nuclei (NI; Felix et al., 1994). The reciprocal connections between the TeO and the NI are the source for a substantial number of cholinergic synapses in the avian midbrain and were attributed an important role in stimulus selection, by separating strong stimuli from their surroundings in a winner-takes-all manner (Wang et al., 2000, 2004, 2006; Marín et al., 2007, 2012). Additionally, tectal cholinergic neurons in intermediate layers were shown to activate local GABAergic neurons, facilitating inhibition in an intra-tectal circuit (Weigel and Luksch, 2014). In general, GABAergic inhibition is often induced *via* cholinergic activation, a principle that is also found in the mammalian SC (Endo et al., 2005). Because of the increasing recognition of the behavioral relevance of the vertebrate midbrain, such networks have recently come into the focus of neurobiological research again (Ito and Feldheim, 2018).

A variety of tectal circuits and their cellular components have been described in detail (reviewed by Luksch, 2003; Wylie et al., 2009), and detailed knowledge of these circuits enables modeling of various aspects of sensory integration, which helps to understand the underlying mechanisms (Lai et al., 2011). In an attempt to further elucidate the tectal circuitry and possible computations in the accessory tectofugal pathway, we here characterize the neurons in tectal layer 9 anatomically and physiologically. These neurons project to the PL (Wild and Gaede, 2016) and present yet another type of radial neurons of the intermediate layers of the TeO. We corroborate their retinal input, identify the modulatory role of GABA and ACh on the neuronal networks comprising these neurons by pharmacological blockade of their receptors, and speculate how these neurons might be incorporated in the accessory tectofugal pathway.

## Materials and Methods

### Animals

The animals used in this study were chicken hatchlings (*Gallus gallus*) of both sexes at ages of just prior to hatching to 4 days after hatching (E21–P4) for slice preparation and subadult, 20–24 day old chickens of both sexes for *in vivo* tracings. Fertilized eggs were incubated at 37°C and 70% humidity and, after hatching, animals were kept at a 12 h dark-light-circle with *ad libitum* access to water and food. All experimental procedures were approved by the local authorities and were conducted in accordance with the National Institute of Health guidelines on the ethical use of animals.

### *In vivo* Tracing

Two adult animals were deeply anesthetized by an intramuscular injection of a 3:1 mixture of Ketamine (50 mg/kg, Inresa Arzneimittel GmbH, Freiburg, Germany) and Rompun (20 mg/kg Xylazin, Bayer, Leverkusen, Germany) and were placed in a modified head holder on a stereotaxic frame (Karten and Hodos, 1967). Under additional local anesthesia, the skin was incised above the skull, the trabecular bone above the optic tectum was opened, and the descending tecto-tegmental tract at the level of the reticular formation underneath the inferior colliculus was identified using stereotaxic coordinates (Kuenzel and Masson, 1988). A micropipette filled with 7.5% (w/v) biotinylated dextran-amine (BDA; MW 3,000; Invitrogen Germany) in phosphate buffer (PB; 0.1 M, pH 7.4) was inserted into the desired location and about 300 nl were injected using a pressure device (Nanoliter 2000, World Precision Instruments, Sarasota, FL, USA). Animals received post-operative treatment and survived for 5–7 days before being sacrificed with an overdose of anesthetics, and subsequent transcardial perfusion with 0.9% normal saline followed by an ice-cold solution of 4% paraformaldehyde (PFA) in PB. Brains were extracted and fixed in 4% PFA in PB for a minimum of 24 h.

### Slice Preparation

Hatchlings were deeply anesthetized by an intramuscular injection of a 3:1 mixture of Ketamine (50 mg/kg) and Rompun (20 mg/kg). Following decapitation, the brain was swiftly extracted from the skull and transferred into oxygenated ACSF (120 mM NaCl, 3 mM KCl, 3 mM MgCl_2_ · 6H_2_O, 23 mM NaHCO_3_, 1.2 mM Na_2_HPO_4_ · H_2_O, 2 mM CaCl_2_ · 2H_2_O, 11 mM D+-glucose) cooled to 4°C. The telencephalon and cerebellum were removed, the two mesencephalic hemispheres were separated midsagittally and embedded in low-melting agar (15 g/l, Sigma-Aldrich, St. Louis, MO, USA) in HEPES buffer (200 mM Saccharose, 3 mM KCl, 3 mM MgCl_2_ · 6H_2_O, 5 mM HEPES). Hemispheres were orientated at an angle differing from the classical transversal plane (Figure 1A) and sliced into 500 μm or 2,000 μm slices using a vibratome (VF-200 Microtome, Precisionary Instruments Inc., Greenville, NC, USA), and collected in oxygenated ACSF. The slices were kept in an interface chamber bubbled with carbogen for recovery for 45–60 min and subsequently kept submerged in continuously oxygenated ACSF at room temperature.

### Tracer Application

Tracer application was performed on slices of 500 μm thickness. Slices were put into a petri dish filled with ACSF, and the application site was delineated with lateral illumination. After draining the ACSF, thin metal rods (200 μm diameter) coated with BDA (3 kD, Molecular Probes, dissolved in A. dest and dried onto the tips) were manually inserted to produce a high-concentration, focal injection into the fiber tract underneath the Stratum album centrale next to the lateral reticular formation (Niederleitner et al., 2017). Slices were returned to ACSF and kept for 4 h at room temperature to allow for tracer transport before fixation in 4% PFA.

### Patch Clamp Recordings

Patch clamp experiments were conducted in accordance to Hamill et al. (1981). The recordings were performed in a custom-built submersion chamber under a fixed stage microscope (Olympus BW50WI, Olympus, Tokyo, Japan) located on a vibration absorbing table (Newport Corporation, Irvine, CA, USA) using an INT-20X (npi electronic GmbH, Tamm, Germany) amplifier and the software WinWCP (John Dempster, University of Strathclyde, Glasgow, UK). Electrodes were pulled on a DMZ-Universal puller (Zeitz-Instruments, Martinsried, Germany) from GB150F-8P glass capillaries (Science Products GmbH, Hofheim, Germany) to a resistance of 3–5 MΩ, filled with patch solution (100 mM K-Gluconate, 40 mM KCl, 10 mM HEPES, 2 mM MgCl_2_ · 6H_2_O, 2 mM Mg-ATP, 1.1 mM EGTA, 0.1 mM CaCl_2_ · 2H_2_0, 13 mM Biocytin-HCl, pH 7.4) and controlled *via* a 3-axis hydraulic micromanipulator (Narishige International, Tokyo, Japan). Approach to the individual cells was conducted under visual control (200× magnification) of the electrode tip using an infrared light source and a DIC-camera and camera controller (C2400, Hamamatsu Photonics, Hamamatsu City, Japan). During the approach, positive pressure was applied to the electrode and once the tip was in contact with the desired cell the pressure was inverted to form a giga-seal. Cells were subsequently opened by short, strong suction and recorded in current clamp mode. The cells were stimulated *via* the patch electrode with 15 consecutive, 100 ms long stimulations of intensities ranging from −150 pA to 200 pA with an increment of 25 pA. After recording, the cells were filled with biocytin by applying current of 0.5 nA for 3 min. The biocytin was then allowed to spread inside the cell for at least 30 min before the slice was fixed in 4% PFA for a minimum of 2 h.

For extracellular stimulation, custom-built electrodes consisting of two twisted coated 50 μm Nickel wires (tip distance >100 μm) stabilized by a glass pipette were inserted into layers 1–2 of the optic tectum. Cells for recording were then chosen at a location slightly offset from the radial axis of the stimulation site to avoid non-synaptic, direct excitation of the cells. Stimulation was performed with 1 ms rectangular pulses at a current well above (150%) the minimal strength that elicited an action potential (AP) for each individual cell 15–300 (μA) using the Isolated pulse stimulus generator Model 2100 (A-M Systems, Sequim, WA, USA). Extracellular stimulation was conducted under physiological conditions (in ACSF) and in calcium-free ACSF (120 mM NaCl, 3 mM KCl, 5 mM MgCl_2_ · 6H_2_O, 23 mM NaHCO_3_, 1.2 mM Na_2_HPO_4_ · H_2_O, 11 mM D+-glucose), as well as under the influence of NBQX (10 μM in ACSF), bicuculline (10 μM in ACSF) and tubocurarine (20 μM in ACSF).

### *In ovo* Transfections

In order to label neurons with GFP we transfected chicken embryos *in ovo* at about stage HH 11–13 following common protocols (Nakamura and Funahashi, 2001; Weigel et al., 2014). The detailed procedure can be seen in video clips published by Yang et al. (2012). Briefly, fertilized eggs (*Gallus gallus domesticus*) were incubated at 38.3°C for approximately 44 h until developmental stages HH11 to HH13. The egg shell was locally stabilized with adhesive tape to prevent cracks. A hole was pinched into the bottom and 4–5 ml albumen was removed. Next, a window was cut into the upper side of the egg exposing the embryo. The embryo was illuminated with blue light to increase the visibility without the need of contrasting substances.

A small volume of plasmid pAcGFP1-F (ClonTech), mixed 1:1 with a Fast Green FCF (0.25%) as dye leaving a plasmid concentration of about 1 μg/μl, was injected into the second brain vesicle that later develops into the mesencephalon. *In ovo* electroporation was performed by applying five voltage pulses (25 V each for 50 ms at 1 Hz; Grass S48 stimulator, Medical Instruments, USA) with gold electrodes placed to each side of the embryo (Genetrodes 45–0115, Harvard Apparatus Inc., Holliston, MA, USA). Afterward, 1–2 ml of chicken-ringer (150 mM NaCl, 5.4 mM KCl, 2.2 mM CaCl_2_, and 2.4 mM NaHCO_3_, pH 7.4) at a temperature of 4° Celsius was pipetted over the embryo to provide cooling in order to recover. Finally, eggs were resealed by means of adhesive tape and incubated until E20–E21.

### Histology

Fixed slices were cryoprotected in 30% sucrose in PB until sunken, subsequently sliced on a sliding microtome (Microme HM 400 E, GMI, New York, NY, USA) in 80 μm sections, and subjected to a DAB protocol with tyramide signal amplification (TSA; Krabichler et al., 2017). After several washes in PB, activity of endogenous peroxidases was blocked in a 3%-H_2_O_2_ solution (in 75% methanol) followed by three more washes in ice-cooled PB. The tissue was then incubated in avidin-biotin peroxidase complex (ABC; Vectastain Elite ABC Kit, Vector Laboratories Inc., Burlingame, CA, USA; 3.2 ml/ml) in phosphate buffered saline (PBS)-Tx100 (0.5%/4% NaCl) for 1 h, washed three times in PB, moved to a solution of 0.0001% biotin-tyramide (IRIS Biotech GmbH, Marktredwitz, Germany) and 0.003% H_2_O_2_ in 0.05 M borate buffer (pH 8.5) for 1 h, washed again three times in PB and incubated in the same ABC-solution as before for 1 h. After washing in PB and acetate imidazole buffer (AIP, 0.175 M acetate, 0.069% imidazole, pH 7.4/6.5), tissue was pre-incubated in a 0.025% diaminobenzidine solution (DAB-buffer tablets for microscopy, Merck KGaA, Darmstadt, Germany) with 1% NiSO_4_ in AIP (pH 6.5) for 5 min and the chromogenic reaction was induced by adding H_2_O_2_ (end concentration 0.0025%) for 3 min. After washing the tissue, the sections were mounted on gelatin-coated slides, counterstained using neutral red, and coverslipped using DPX (DPX Mountant for Histology, Sigma-Aldrich GmbH, Steinheim, Germany). The brains from *in vivo* tracing experiments were treated in the same way but were sliced in 50 μm sections and the TSA steps were omitted.

For the visualization of transfected cells, animals were decapitated under deep anesthesia, brains were isolated and immediately fixed in 4% PFA (in PBS: 0.023 mM NaH_2_PO_4_, 0.08 mM Na_2_HPO_4_, pH 7.4) for at least 24 h. They were transferred to a sucrose solution (30% sucrose in PB) for at least overnight, cryosectioned into 100 μm thin sections, counterstained with DAPI and mounted on microscope slides in n-Propylgallat (0.2%, diluted in DMSO, Glycerol and PB).

### Immunohistochemistry

Slices from the tracing experiments were fixed in 4% PFA in PB for a minimum of 2 h, transferred into 30% sucrose (w/v in 0.1 M PB) overnight for cryoprotection and then resectioned in 30 μm thin sections (Microm HM440E). The sections were rinsed in PBS (0.1 M PB with 0.75% NaCl, three times for 5, 10 and 15 min) followed by an incubation with a blocking solution (3.5 h at RT; 0.1 M PB, pH7.4) containing 0.5% bovine serum albumin (BSA, Roth, Cat# 0163.2), 5% normal goat serum (NGS, Linaris S-1000, Cat# ADI-20011-100) and 0.5% Triton X-100 (Tx100, Fluka). The primary antibody against neurofilament 200 (NF200, rabbit polyclonal, Sigma-Aldrich, Cat. #N4142, AB_477272) was diluted (1:1,000) in 0.1 M PB containing 1% BSA and 5% NGS. The sections were incubated with the primary antibodies overnight at 4°C. The specificity of the epitope was shown in an earlier study (Lischka et al., 2018). After washing (3×), sections were incubated in the secondary antibody (Alexa Fluor 488 goat anti-rabbit, 1:500, Thermo Fischer Scientific, Waltham, MA, USA, Cat# A11010, RRID: AB_10584649) overnight at 4°C. Afterward, sections were washed again and incubated in Alexa 546-conjugated Streptavidine (1:500 in 0.1 M PBS with 0.5% Tx100, #S11225, Molecular Probes) for 2 h at RT. The sections were mounted on microscope slides in n-Propylgallat.

### Microscopy and Reconstruction

Transfected neurons were photographed with an epifluorescence microscope (Olympus BX3-CBH, Olympus, Germany) equipped with a CCD-camera (XM-10, Olympus, Germany) and appropriate filters (DAPI: Ex 350/50 nm, EM 460/50 nm, BS 400 LP; eGFP: Ex 470/40 nm, EM 525/50 nm, BS 495LP nm; AHF Analysentechnik, Tübingen, Germany). Image stacks in z-direction were taken and deconvoluted with a Wiener-kernel. Neurons were reconstructed manually using the freeware NEUTUBE (Feng et al., 2015). To test for the homogeneity of the reconstructed neurons, we performed a Sholl analysis, a method used for quantitative assessment of neuronal arborization (Sholl, 1953). Here, we count how often the reconstructed neurons cross three-dimensional spheres of evenly spaced radii of 1 μm with a midpoint at the soma of each neuron. Sholl analyses were performed in ImageJ using the “Simple Neurite Tracer” plugin (Longair et al., 2011). Data were further processed and normalized to the distance between the outer border of the tectum (L1) and the borders of the layers 9/10 in MATLAB (MATLAB_R2017b, The MathWorks, Natick, MA, USA). Manual reconstructions of intracellularly filled neurons were done with a Leitz Dialux 20 (Leica Microsystems GmbH, Wetzlar, Germany) microscope equipped with a camera lucida. Figures were then assembled with the use of the software Affinity Photo (Serif, Nottingham, UK).

To analyze the localization of NF200 on the neurons of interest, we took fluorescence images stacks with a confocal laser scanning microscope (Olympus FV1000/IX81, Olympus, Germany) using a 25× or a 40× objective. Image stacks were imported to FIJI (ImageJ 1.52i). Here, fluorescence channels were separated, filtered with a mean 3D filter (2 2 2) and adjusted for contrast and brightness. The stacks were reduced and focused using the “stack focuser” plugin provided by Michael Umorin. Afterwards, channels were merged to obtain an overlaid image of the retrogradely labeled neurons and NF200.

### Statistical Analysis

Processing of physiological data, statistical analysis as well as data plotting was performed in MATLAB. Raw data were saved in the preset WinWCP data format (.wcp) and imported into MATLAB by the use of the WinWCP MATLAB Importer (MathWorks File exchange, provided by D. Jäckel, Zurich, Switzerland). For calculation of the membrane resistance, the relation of the membrane voltage at a point near the end of the duration, where a steady state could be expected, to the applied current was plotted. A linear fit to the data in the current range of −100 pA to 0 pA was performed. The slope of the partial regression line is the membrane resistance. The time constant τ was calculated for the same current range graphically as the time to pass after the stimulation until the cell reaches 1/e of the total hyperpolarization.

From electrostimulation experiments, one representative recording per cell was used for analysis. The stimulation was recorded on a second channel and identified by the findpeaks function. To calculate both latency and duration of depolarization after an electrostimulation, we set a threshold at −50 mV. Latency was then calculated as the time difference between the stimulation and the first passing of the threshold, time to the first AP as the time difference between stimulation and peak of the first AP (found by findpeaks function), number of APs as the number of peaks with a minimal height of −20 mV and duration as the time between the first passing of the threshold before the AP and the first time the depolarization following the AP falls under the threshold. For analysis of cells treated with calcium free ACSF and pharmaceuticals, one recording just before start of the treatment (control), one recording at 15 min after begin of the treatment (treatment) and one recording at 20 min after the start of washing (wash) was used.

Data were tested for normal distribution by the Kolmogorov–Smirnov test (KS-test in MATLAB) and, as most of the data did not follow this distribution, all comparisons were performed using the Mann–Whitney *U* test (rank sum in MATLAB) or the Friedman’s test in case of repeated measures. If data were normally distributed, data were displayed as mean ± standard deviation. In all other cases, values in the text were displayed as “median (range).”

## Results

### Cells Projecting to the Lateral Pontine Nucleus

**Figure 2:**
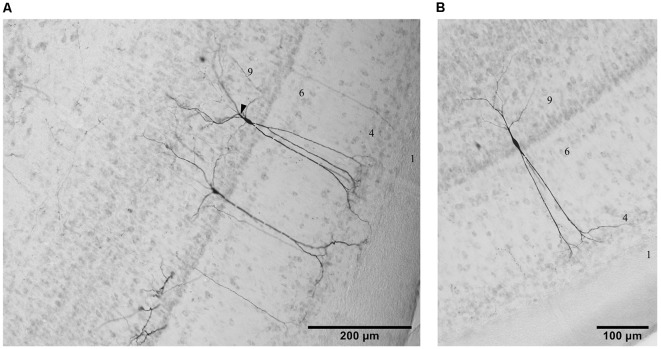
Photomicrographs of three examples of cells in layer 9 from the lateral **(A)** and the ventral **(B)** aspect of the TeO labeled by tracing experiments in the descending tract. Lamination of the TeO is indicated by corresponding numbers. Arrow head in **(A)** indicates a characteristic branching of basal dendrites in some layer 9 neurons.

In the two animals where tracer application was located in the tecto-tegmental tract at the level of the reticular formation underneath the inferior colliculus, a variety of cell types throughout the midbrain was labeled. Within the TeO, numerous cell types in different layers were retrogradely filled. As these cell types have already been reported in Reiner and Karten (1982) as neurons belonging to the ipsilateral tecto-pontine and tectospinal tract, we will not give a detailed account of the morphology and the laminar location of these cells, but focus on the most conspicuous cell type that was located in layer 9 and consisted of sparsely distributed small-sized bipolar cells (Figure 2). These cells had fusiform somata and were oriented radially. Two to three apical dendrites starting near the soma were passing straight through layers 9–5 and further arborizing in layers 4–2. The basal dendrites started branching close to the soma and ramified in layers 9 and 10, giving the cells an almost pyramidal appearance. Although the labeling of somatic and dendritic structures was very clear, axons could not always be distinguished as such (see below).

### Morphology of Layer 9 Neurons

**Figure 3:**
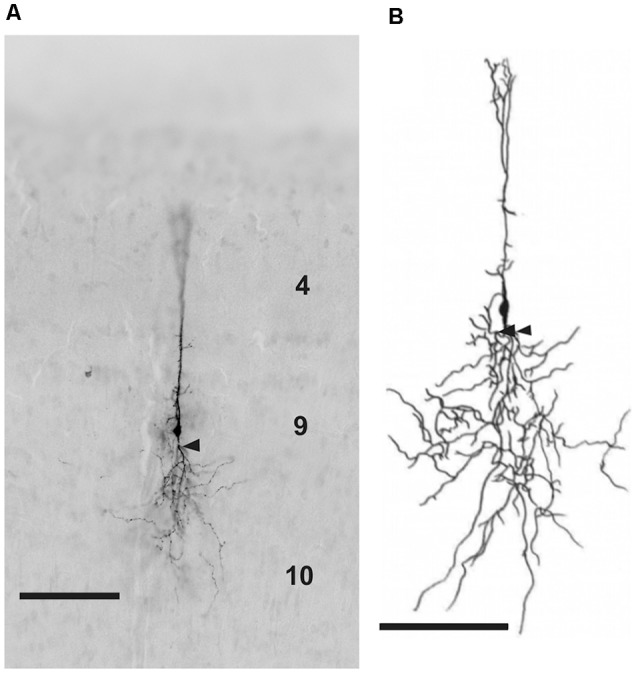
**(A)** Photomicrograph and **(B)** reconstruction of a neuron in layer 9 of the TeO labeled with biocytin after whole-cell patch recording. Layers are marked by corresponding numbers. Scale bars 100 μm. Arrow heads indicate the characteristic branching of basal dendrites in some layer 9 neurons.

To characterize the neurons of layer 9 further, a total of 116 cells in layer 9 were labeled intracellularly in 96 slices of 29 chicken hatchlings. Only neurons with a seemingly complete dendritic organization were further analyzed, leaving 29 fully intact cells. All of these neurons had a fusiform soma and extended dendrites in both directions (Figure 3). While some features of the neurons (e.g., presence or absence of a major branching of the basal dendrite close to the soma) appeared to justify the differentiation into subclasses, statistical analysis on transfected neurons (see below) did not support this classification. Thus, neurons will be described as a single type. The cells labeled in layer 9 by tracing experiments as well as the results of *in ovo* transfections described in a later paragraph also corroborated this overall pattern.

**Figure 4:**
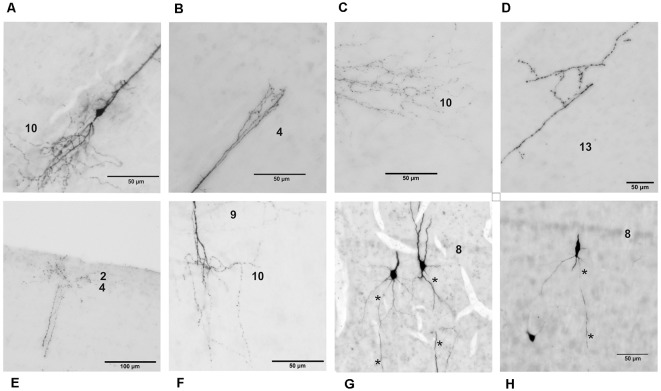
Detailed photomicrographs of various parts of chicken OT layer 9 neurons. **(A)** Soma of a typical neuron. **(B)** Arborization of the apical dendrite in layer 4. **(C)** Ramification of basal dendrites in layer 10. **(D)** Fine processes of basal dendrites in layer 13. **(E)** Fine arborization of the apical dendrite in layers 2–4. **(F)** Dendritic processes in layers 9 and 10. **(G)** Extended depth of field image of cells with axon origin at the basal soma. **(H)** Extended depth of field image of a cell with axon origin at the basal dendrite. Axons are indicated by the asterisks.

**Figure 5:**
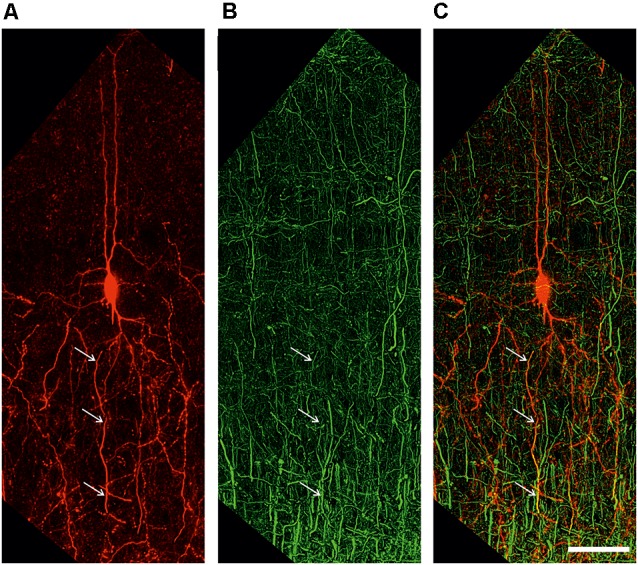
Extended depth of field image of a retrogradely labeled neuron **(A)**, an antibody labeling against the axonal marker NF200 **(B)**, and the overlay of both channels **(C)**. White arrows indicate colocalization. Scale bar 50 μm.

Cells in layer 9 of the chicken optic tectum (Figure 4) have fusiform somata with 1–3 apical primary dendrites and one primary basal dendrite, which in 60% (17/29) had a prominent branching point close to the soma. Laterally oriented dendrites occur only sparsely; accordingly neurons usually have a total of two to up to four primary dendrites. Apical dendrites usually run straight through layers 7–5 and arborize primarily in layers 4–2. The majority of cells (approximately 80%) had additional short processes in layers 1, 5, 6 and 8. After branching, the basal dendrites form many local processes in layers 9 and 10 and a few minor processes in layer 13. Laterally extending dendrites are much smaller and end very locally in layers 8 and 9. Axons can only be distinguished from dendrites in rare cases, but when observed, run straight in basal direction towards the ventricle, with a sharp turn in layer 15. To better understand axonal organization, we performed double labeling of retrogradely labeled layer 9 neurons with immunohistochemistry against neurofilament 200, an abundant protein in axons (Figure 5). Based on these findings, we could confirm that axons originate from either the basal side of the soma or from a basal dendrite close to the soma (Figures 4G,H). With a cross section size of 42.53 (69.47) μm^2^, cell somata are of rather small size, and have a median length of 9.87 (6.75) μm and a width of 5.49 (5.62) μm (as data are not always normally distributed, values are given as median and range). The soma is located at a distance of 42.73 (48.61) μm from layer 8. Maximal dendritic spread in the radial dimension is 432.00 (443.44) μm, and 199.00 (423.20) μm in the lateral dimension.

**Figure 6:**
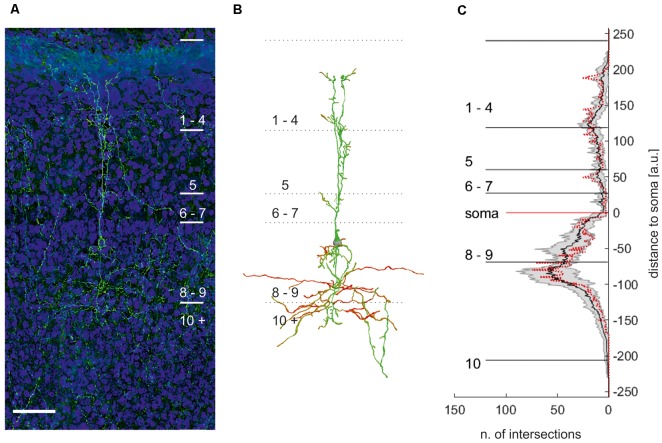
Maximum projection of an eGFP expressing neuron in layer 9 of the optic tectum **(A)**. **(B)** Manual reconstruction of the neuron with soma location in layer 9 in **(A)**. Different colors code for the direction of the neurite. Note: reconstruction allows distinguishing between different cells in this area. Scale bar = 75 μm. **(C)** Plot of the mean numbers ± standard deviation (*n* = 7) of intersections per μm with respect to the distance to the soma. Red dotted line: sholl analysis of the neuron depicted in **(B)**.

As both tracing and intracellular staining may still miss cellular details due to technical reasons, we reconstructed eGFP-expressing neurons in layer 9 (Figures 6A,B). In these genetically labeled cells, we further performed a statistic analysis of the dendritic organization (Sholl analysis) to assess the homogeneity of the population.

All neurons had a similar morphology comparable to intracellular fillings, with their somata located in the apical part of layer 9. We observed neurites apical of the soma reaching up to layer 2 and basal of the soma projecting into layer 10. The apical neurites ramified in layer 5 into up to three larger, parallel branches, with a columnar shape of about 40–50 μm width. They further exhibited smaller branches in all superficial layers but mostly in the apical part of layer 5 (layer 5a), 4, and 2. Originating basally at the soma, we found short, fine neuritic arborizations in layer 9. In addition, broadly extending (200 μm), fine neurites start at the soma and project mainly into layer 10a. Sometimes, fine processes extended into the deeper layers but could not be traced to their end. Figure 6C shows a Sholl analysis of the number of intersections at a normalized distance from the cell body (bin size = 1 μm; *n* = 7). The small standard deviation of the Sholl plot indicates that the morphology of layer 9 neurons is rather uniform. We thus concluded that layer 9 neurons in the chick consist of only one cell type.

### Physiological Parameters of Layer 9 Neurons

Cellular properties were measured in stable whole-cell recordings by applying a series of current injections with increasing strength (*n* = 45). All measured properties were normally distributed and presented as mean ± standard deviation.

**Figure 7:**
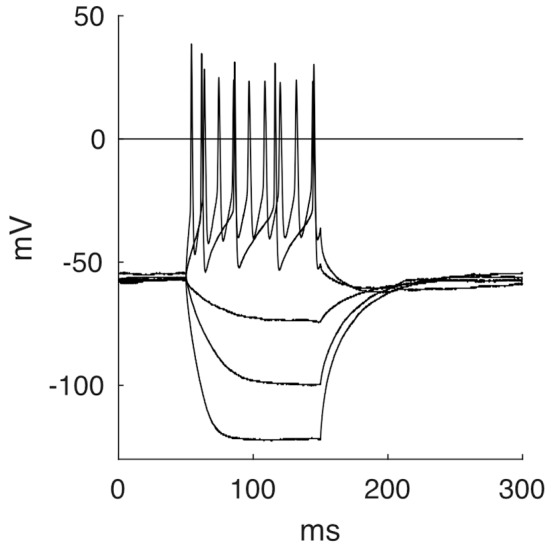
Five selected response traces of the stepwise stimulation with currents of −150 pA, −75 pA, −25 pA, 75 pA and 200 pA.

By applying negative currents, cells showed hyperpolarizations of hyperbolic shape until reaching a steady state, followed by a repolarization with occasional overshoot of the baseline. These rebounds appeared to be caused by increased sodium influx through leakage channels during hyperpolarization and not by specific Ih-channels, as the latter responds with a characteristic voltage sag not seen here (Luksch et al., 2001). Most cells responded to depolarizing current pulses (over a threshold of about 50 pA) by firing a continuous and regular series of APs throughout the stimulus duration (Figure 7, 37 out of 45). Seven cells showed a phasic response and one cell was firing only one AP even at higher depolarizing currents. Usually, we observed higher AP frequencies upon increasing current. The neurons had a resting membrane potential of −58.8 mV ± 4.3 mV. They had to be depolarized to a membrane potential of −34.8 mV ± 4.5 mV before evoking an AP with a half maximal width of 1.6 ms ± 0.3 ms. The mean membrane resistance for layer 9 neurons was 859.3 MΩ ± 231.6 MΩ. The membrane time constant τ was 50.2 ms ± 17.5 ms. Firing behavior was not correlated to a particular passive property.

### Retinal Input

**Figure 8:**
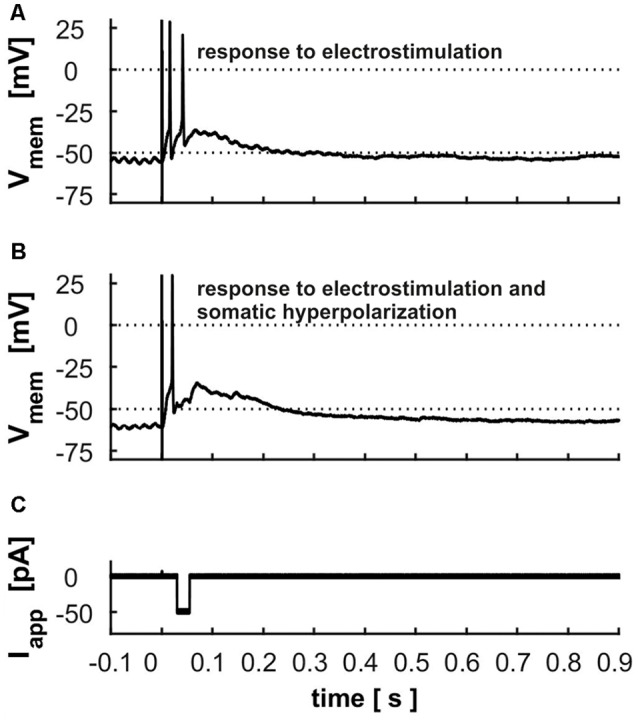
Electrostimulation in superficial layers **(A)** response of a layer 9 cell to the electrostimulation; dashed line indicates the threshold used for calculation of the latency and duration of depolarization **(B)** response of the same cell to the electrostimulation with a hyperpolarization of −50 pA during the depolarization phase (displayed in **C**).

Cells usually responded to electrical stimulation of retinal input in layer 2 with 1–2 APs riding on a long-lasting depolarization (Figure 8A). Fifty-two cells were tested regarding their latency, the time to the first AP, the number of APs and the duration of the depolarization following the AP. On average, response latency was found to be 13.4 ms ± 8.0, the time to the first peak of an AP 17.9 ± 9.4 ms, the number of triggered APs 1.5 ± 0.8 and the duration of depolarization 112.1 ms ± 94 ms. To test whether the long-lasting depolarization is caused by intrinsic cellular properties or due to network activity, we applied negative currents with a variable delay after electrostimulation to disrupt intrinsic activity by hyperpolarization. However, cells rebounded to the previous depolarized state even after strong hyperpolarization (Figures 8B,C).

### Pharmacological Studies

**Figure 9:**
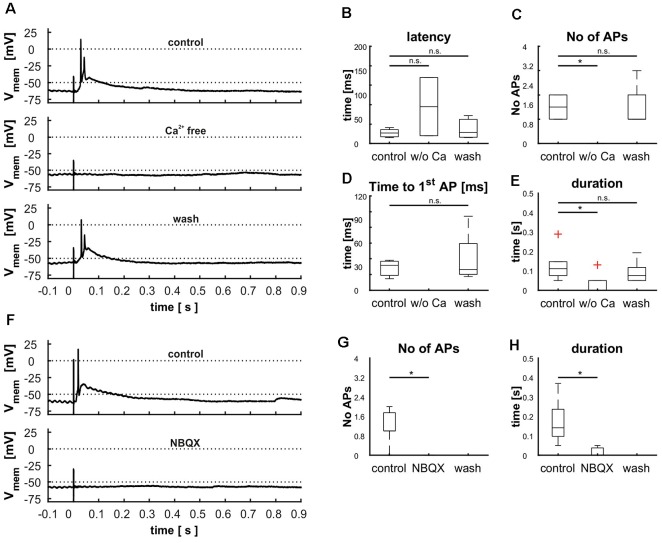
Demonstration of retinal input to cells in layer 9 *via* glutamatergic synapses by treating cells with calcium free ACSF **(A–E)** and blocking AMPA receptors with NBQX **(F–H)**. **(A)** Cellular response before, during and after the treatment with calcium free ACSF, **(B)** effect of calcium free ACSF on the latency [control: 21.6 ms (20.6 ms), Ca^2+^ free: 75.9 ms (119.8 ms), wash: 22.8 ms (45.2 ms), *p*(control vs. Ca^2+^ free) = 0.59, *p*(control vs. wash) = 0.94], **(C)** the number of action potentials [APs; control: 1.5(1), Ca^2+^ free: 0 (0), wash: 1(2), *p*(control vs. Ca^2+^ free) = 0.01, *p*(control vs. wash) = 0.95], **(D)** the time to the first [AP; control: 31.7 ms (23.6 ms), Ca^2+^ free: n.e., wash: 26.3 ms (76.8 ms), *p*(control vs. Ca^2+^ free) = n.e., *p*(control vs. wash) = 0.75], and **(E)** the duration of depolarization [control: 0.11 s (0.23 s), Ca^2+^ free: 0 s (0.13 s), wash: 0.07 s (0.14 s), *p*(control vs. Ca^2+^ free) = 0.03, *p*(control vs. wash) = 0.95). **(F)** Cellular response before and during the treatment with NBQX **(G)** effect of NBQX on the number of APs [control: 1 (2), NBQX: 0 (0), wash: n. e., *p*(control vs. NBQX) = 0.01] and effect of NBQX on the duration **(H)**, [control: 0.14 s (0.32 s), NBQX: 0 s (0.05 s), wash: n. e., *p*(control vs. NBQX) = 0.14]. Significance (*p* < 0.05) is indicated by asterisks, n.s. = not significant, n.e. = not existent.

To rule out direct stimulation of the cell’s dendrites, we blocked synaptic transmission by application of calcium-free ACSF (*n* = 6). Reliably, AP generation was eliminated (*n* = 6, Figures 9A,D), corroborating the synaptic nature of the electrical stimulation. However, in three recordings small EPSPs were visible that correlated only in one case with the stimulus. Reintroduction of calcium showed that cells did not suffer damage by the treatment as all measured parameters reached the same levels as before (Figures 9A–E).

Next, we tested whether synaptic transmission could be suppressed with a glutamate antagonist (NBQX, *n* = 7). Again, incubation of the slices with NBQX completely abolished AP generation (Figures 9F–H). Similar to the application of calcium-free ACSF, small EPSP remained in five cells indicating a slight direct stimulation in a few cases. Washout of the pharmaceutical could not be achieved in a reasonable amount of time, probably due to its high affinity to the AMPA receptor.

**Figure 10:**
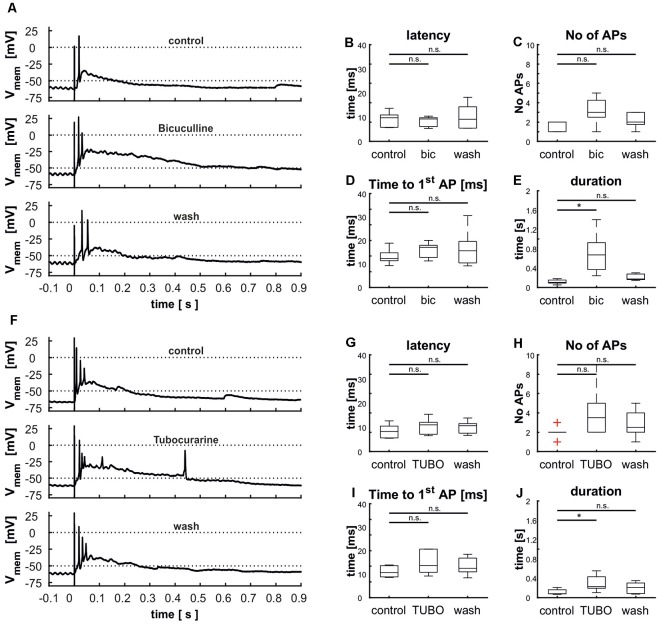
Modulation of cellular response by γ-aminobutyric acid (GABA) and acetylcholine (ACh) shown *via* the blockage of GABA_A_ receptors with bicuculline **(A–E)** and nicotinic ACh receptors with tubocurarine **(F–J)**. **(A)** Cellular response before, during and after the treatment with BIC, **(B)** effect of BIC on the latency [control: 9.8 ms (8.0 ms), BIC: 9.3 ms (5.1 ms), wash: 9.2 ms (12.8 ms), *p*(control vs. BIC) = 0.41, *p*(control vs. wash) = 0.80], **(C)** the number of APs [control: 2 (1), BIC: 3 (4), wash: 2 (2), *p*(control vs. BIC) = 0.08, *p*(control vs. wash) = 0.38], **(D)** the time to the first AP [control: 12.3 ms (9.6 ms), BIC: 17.1 ms (8.8 ms), wash: 15.6 ms (21.5 ms), *p*(control vs. BIC) = 0.80, *p*(control vs. wash) = 0.95], and **(E)** and the duration of depolarization. [control: 0.10 s (0.13 s), BIC: 0.67 s (1.16 s), wash: 0.17 s (0.16 s), *p*(control vs. BIC) = 0.01, *p*(control vs. wash) = 0.25], [**F**] cellular response before and during the treatment with TUBO, **(G)** effect of TUBO on latency [control: 8.3 ms (7.2 ms), TUBO: 11.1 ms (8.9 ms), wash: 10.7 ms (7.2 ms), *p*(control vs. TUBO) = 0.14, *p*(control vs. wash) = 0.46], **(H)** the number of APs [control: 2 (2), TUBO: 3.5 (7), wash: 2.5 (4), *p*(control vs. TUBO) = 0.08, *p*(control vs. wash) = 0.68], **(I)** the time to the first AP [control: 10.6 ms (5.3 ms), TUBO: 13.3 ms (11.5 ms), wash: 12.5 ms (10.1 ms), *p*(control vs. TUBO) = 0.19, *p*(control vs. wash) = 0.66], and **(J)** the duration of depolarization [control: 0.08 s (0.14 s), TUBO: 0.23 s (0.45 s), wash: 0.21 s (0.28 s), *p*(control vs. TUBO) = 0.01, *p*(control vs. wash) = 0.11]. Significance (*p* < 0.05) is indicated by asterisks, n.s. = not significant.

The tectal network contains a multitude of elements that exert both local and global modulation. To assess the contribution of inhibitory input upon the cellular response elicited by stimulation of the retinal afferents, we blocked GABA_A_ receptors with the competitive antagonist bicuculline (BIC; *n* = 5). This pharmacological inhibition led to an extensively increased duration of depolarization [103 ms (130 ms) vs. 671 ms (1,159 ms); *p* = 0.008], which was reduced to initial value after removal of the pharmaceutical [173 ms (157 ms); *p* = 0.056; Figures 10A,E]. Latency [control: 9.8 ms (8.0 ms); bic: 9.3 ms (5.1 ms; *p* = 0.69); wash: 9.2 ms (12.8 ms; *p* = 0.84)] and time to the first AP [control: 12.3 ms (9.6 ms); bic: 17.1 ms (8.8 ms; *p* = 0.69); wash: 15.6 ms (21.5 ms; *p* = 0.84)] were not affected by the experiment (Figures 10B–D); we observed a slight increase in the number of APs [control: 2 (1); bic: 3 (4; *p* = 0.095); wash: 2 (2; *p* = 0.397)], which was however not significant (Figure 10D).

Another common type of modulation in the tectum acts *via* ACh. To examine its influence on layer 9 cells, we applied a competitive blocker of nicotinic ACh receptors (tubocurarine, TUBO) and repeated electrical stimulations (Figure 10F, *n* = 6). Treatment resulted in an increased duration of depolarization (Figure 10J); this effect disappeared upon removal of the pharmaceutical [control: 80 ms (143 ms); TUBO: 226 ms (452 ms; *p* = 0.01); wash: 201 ms (278 ms; *p* = 0.13)]. The other parameters were not influenced, but analogously to the treatment with BIC the number of APs was increased slightly but not significantly [Figures 10G–I; control: 2; bic: 3.5 (7; *p* = 0.08); wash: 2.5 (4; *p* = 0.39)]. These prolonged depolarizations could again not be interrupted by hyperpolarization *via* application of negative current through the patch electrode (data not shown).

## Discussion

### Radial Neurons in Layer 9 of the Avian Optic Tectum

Following tracer injections into the tecto-tegmental tract, we found retrogradely labeled neurons in layers 9, 10 and 13 of the ipsilateral TeO. While axonal structures could not be observed in many of these neurons, the nature of their labeling argues for a methodological reason. It is possible that axonal projections are thin and not easily be detected with a standard DAB visualization; here, the use of a TSA intensification protocol might yield better results (Krabichler et al., 2017).

Reiner and Karten (1982) studied the ipsilateral tectopontine-tectoreticular descending pathway in pigeons and found tectal neurons in layers 8–15, whereas the other major descending pathway, the crossed tectobulbar pathway, only received input from layers 13–15. Wild and Gaede performed retrograde tracing experiments in the PL of zebra finches and also found tectal neurons in layers 8–15, including radial, fusiform neurons in layer 9 that resemble the population characterized here (Wild and Gaede, 2016). The application site in our experiments thus appears to be part of the ipsilateral tectopontine-tectoreticular pathway, as fibers running ventrally were labeled, and the PL seems a likely target for the projections of layer 9 neurons. Projecting to the DLPc/Uva, the PL connects neurons in layer 9 to the accessory tectofugal pathway originally described by Gamlin and Cohen (1986).

Although the cells characterized here have not been described in detail earlier, the existence of numerous radially oriented cell types in the TeO especially in the intermediate layers is well known (Cajal, 1911; Gamlin and Cohen, 1986; Wild, 1989; Woodson et al., 1991; Vega-Zuniga et al., 2014). We here describe neurons in layer 9 with a high overlap in their characteristics with other radial neurons. This includes the relatively small soma size, the presence of only few primary apical and basal dendrites, and the extension of the apical dendrite to retinorecipient layers. The arborization of apical dendrites in layers 2–4 indicates retinal input in these layers, with the cadherin-7-positive population of retinal ganglion cells being a candidate as this cell type projects to layer 4 (Yamagata et al., 2006). As only few cells in layer 9 of the TeO are cadherin-7 positive (Yamagata et al., 2006), this finding might help to further delineate the connectivity in future studies. In the mouse, cadherin-7 regulates lamination of directionally selective retinal ganglion cells (Duan et al., 2018); it will be interesting to see whether comparable circuit functions can be established in the chick too. Other cell types with dendritic arborization in superficial layers such as the “shepherd’s crook” neurons in layer 10 (Woodson et al., 1991) and stratum griseum centrale (SGC) neurons in layer 13 (Luksch et al., 2001) have also been shown to receive retinal input. The role of basal dendrites ramifying in layer 9–13, however, cannot easily be delineated, as input to these layers may come from various sources including the NI, auditory midbrain, and even descending input from the forebrain (Wang et al., 2006; Wylie et al., 2009; Niederleitner et al., 2017). In general, the parallels in morphology of radial tectal neurons suggest that they also share functional properties. At the slender apical dendrites that have little lateral extension, these radial cells likely receive visual input from a narrow angle of the spatial map which, taken into account that the retinal afferents do not spread far laterally, could yield a high spatial resolution. Accordingly, receptive fields (RFs) recorded in the superficial layers of chicken (down to approximately layer 9) are usually small (3.8 degrees of visual angle), whereas the deep layers have RFs with an average of 10 degrees visual angle (Verhaal and Luksch, 2013). On their basal dendrites, however, additional input is integrated much more coarsely, as the basal dendritic fields extends much further laterally.

The fact that in the intracellular fills axons could not always be detected could indicate that layer 9 neurons are at least partly a population of local interneurons. As TSA was used in the detection of intracellular labeled cells, it is unlikely that axons were missed due to being too thin. However, the missing axon likely has a methodological explanation, as the patched cells were all located quite superficially, and axons thus may have often left the slice boundaries. This interpretation is corroborated by further anatomical analysis that did not reveal different cell types in the genetically labeled neurons. The low retrieval of axons in intracellular fills has also been observed for other cell types in the chicken TeO, e.g., in the “shepherd’s crook” neurons that project to the isthmic nuclei (Lischka et al., 2018) and “vine” neurons that project to the nucleus geniculatus lateralis pars ventralis (Vega-Zuniga et al., 2014).

### Physiology of Neurons in Layer 9

As somata of cells in layer 9 were rather small, high membrane resistances could be expected, which also explain the low current threshold to trigger APs. The regular spiking pattern of most recorded neurons resembles that of SGC-II neurons (Luksch et al., 2001) and other cells in the optic tectum of pigeons (Hardy et al., 1987). While it is rather common, it is not the only form of response to somatic current injection observed in the TeO, as SGC-I neurons show a characteristic chattering behavior (Luksch et al., 2001).

As retinal ganglion cell axon terminals extend over the entire surface of the TeO, retinal input is easy to simulate by applying current with a small bipolar stimulation electrode inserted into superficial layers, activating only a few retinal afferents. The usual response, a steeply rising membrane potential forming an AP riding on a prolonged depolarization, resembles the response of SGC-II neurons to the same type of stimulation (Luksch et al., 2001). We could demonstrate that the depolarization following the AP could not be suppressed by somatic hyperpolarization. We thus conclude that the long-lasting depolarization results from activity in the network. As the stimulation electrode stimulated a limited, but nevertheless extended set of retinal afferents, it is likely that several tectal cells were activated and led to the network activity observed.

### Synaptic Transmission

The dendritic arborization of layer 9 neurons in superficial layers and the latency to electrical stimulation argued for a synaptic nature of the input, and not a direct stimulation of the cell’s dendrite. This was corroborated by stimulation in calcium-free ACSF, which abolished responses in almost all cases. In addition, we could show that blocking of AMPA receptors also abolished the cellular response to retinal stimulation. Glutamate is the major neurotransmitter mediating input from retinal ganglion cells in many vertebrates (Canzek et al., 1981; Binns and Salt, 1994). The finding that cellular responses could be suppressed by the AMPA receptor inhibitor NBQX clearly identifies the synapse between RGCs and layer 9 neurons as glutamatergic, with characteristic response latencies of about 13.4 ms ± 8 ms. This latency is in the range of other cell types in the same preparation [Shepherd crook cells: 6.9 ms ± 1.3 ms (Meyer et al., 2008), SGC type 1 cells: 11 ms ± 2 ms, type II cells: 14 ms ± 6 ms (Luksch et al., 2001)]. Whether the retinal input onto layer 9 neurons is monosynaptic or not cannot be conclusively answered without electron microscopy, but seems to be very likely.

### Modulation Mediated by GABA and Acetylcholine

Modulatory elements exist in the TeO both locally and globally and are often mediated by the neurotransmitters GABA and ACh. One source of global modulatory connections with the TeO are the isthmic nuclei, a network that establishes a winner-takes-all computation that relies on both ACh and GABA release in the TeO (Felix et al., 1994; Wang et al., 2006; Goddard et al., 2012; Marín et al., 2012; Mysore and Knudsen, 2013). Additionally, local inhibitory networks, for example the GABAergic horizontal cells in layers 4 and 5, and local cholinergic modulations of inhibitory networks have been described (Tömböl and Németh, 1999; Luksch and Golz, 2003; Weigel and Luksch, 2012; Weigel et al., 2014). As modulation through GABA and ACh thus appears to be dominant in the TeO, we focused on these transmitters.

Inhibition of the GABA_A_ receptor resulted in a strongly increased duration of depolarization after retinal stimulation that was reduced to initial values after washing. As the excitation of layer 9 neurons by retinal input includes network activation as shown before, we conclude that this network activity is under GABAergic suppression under normal physiological conditions. The cellular origin of this inhibition is difficult to determine, as GABAergic cells are present in all layers throughout the TeO and in various nuclei of the midbrain (Domenici et al., 1988; Veenman and Reiner, 1994). Local GABAergic inhibition might, therefore, be one plausible explanation, as it has been suggested for neurons in intermediate layers before (Weigel and Luksch, 2012). One potential source are GABAergic horizontal cells in layer 4 (Tömböl, 1998), similar to the situation in SGC neurons, where synaptic transmission from retinal efferents is restricted by horizontal layer 5b neurons within glomeruli around the synapse (Luksch and Golz, 2003). The feed-forward inhibition established by this circuit might explain the slight increase of AP number under the influence of BIC. Such feed-forward inhibition is a prevalent mechanism to increase temporal resolution (Roberts et al., 2013).

Another possible source of inhibitory neurons is the IMC. The connection of the isthmic nuclei to the TeO was largely intact in all slices and IMC has a GABAergic projection to layers 10–12 (Wang et al., 2004). GABA_A_ receptors are strongly expressed in the intermediate layers of the avian tectum. However, layer 9 neurons have not been explicitly shown to express the receptor and the layer was only weakly labeled in *in situ* and immunohistochemical studies (Glencorse et al., 1991; Veenman et al., 1994). It is therefore conceivable that GABA does not act on the neurons examined in this study directly, but rather on different neurons with excitatory effect, thus leading to the network characteristic observed.

Blocking of nicotinic ACh receptors resembled the effect of GABA receptor blocking and led to a prolongation of the EPSP following the AP after retinal stimulation. This resemblance argues for the close interaction of the two systems, which has been described in both mammalian and avian studies before (Endo et al., 2005; Weigel and Luksch, 2014). As the absence of cholinergic activation seems to suppress inhibition completely, we conclude that GABAergic inhibition is activated *via* ACh.

In our study, we could not determine the cellular elements that give rise to these cholinergic and GABAergic effects. Thus, we cannot delineate whether these modulations are directly elicited by retinal input, or whether the extended circuitry in the TeO and beyond is involved. As for the cholinergic input neurons, both the NI, where high levels of acetylcholinesterase immunoreactivity and reciprocal connections to the tectum were observed (Wang et al., 2006) or the abundant cholinergic cells in the layer10 of the tectum could play a role (Sorenson et al., 1989; Medina and Reiner, 1994). Weigel and Luksch (2014) showed that disinhibition could not be achieved by removal of the isthmic tract and proposed an entirely local circuit to be responsible for the ACh-mediated GABAergic inhibition encountered. Thus, tectal processing would be less dependent on elements beyond the TeO, but rather on the activation *via* sensory input. While it is obvious that ACh acts on GABAergic cells, the manner of transmission remains unclear. Modulation of neurotransmitter release at the presynapse, postsynaptic activation of voltage-gated channels and nonsynaptic modulation of membrane resistance are all known effects mediated by the nAChR (Dani and Bertrand, 2007).

### Layer 9 Neurons and the Accessory Tectofugal Pathway

In mammals, the homologous structure of the TeO, the SC, is considered a center for multimodal integration (Meredith et al., 1992; Basso and May, 2017) and the avian tectum has long been taken as a model system for audio-visual integration (Knudsen, 1982; Wylie et al., 2009). While the function of the tectofugal pathway has been discussed in various studies (e.g., Marín et al., 2003), the role of the accessory tectofugal pathway remains obscure. However, as this pathway includes the TeO, the DLPc/Uva and the PL which have all been shown to compute multimodal information, multimodality seems to be firmly integrated into the accessory tectofugal pathway. DLPc/Uva has even been proposed as one example where trimodal integration takes place (Wild, 1994).

How multimodal integration is accomplished in this pathway is so far unclear. While some neurons in the TeO presumably process both visual and auditory input (Lischka et al., 2018), we have here only demonstrated visual input to the radial layer 9 neurons of the TeO that project to the PL. Whether additional auditory or somatosensory input is also relayed by the layer 9 cells remains to be investigated. Auditory input could also reach the PL directly as it has been shown in cats (Aitkin and Boyd, 1978). By which way the somatosensory input is fed into the accessory tectofugal pathway is unknown; even though the auditory and the somatosensory systems are closely connected (Wild, 1995), any detailed information on somatosensory input to the tectum are so far lacking.

### Layer 9 Cells as Modulatory Neurons

The avian TeO contains a variety of radial neurons with distinct characteristics such as “shepherd’s crook” neurons, “vine” neurons or the layer 9 neurons characterized here. Processing of high-resolution visual information seems to be a shared primary function of this neuron type. *In vivo* studies of the avian tectum have shown increasing RF sizes with increasing distance from the tectal surface, with RFs of a few degrees in the superficial layers (Jassik-Gerschenfeld and Guichard, 1972; Verhaal and Luksch, 2013). Neurons recorded there often respond maximally to flashed high-contrast stimuli, in contrast to cells in the deep layers that have wide RFs and responded to motion and apparent motion (Frost et al., 1988; Luksch et al., 1998; Verhaal and Luksch, 2016b). While in our *in vitro* preparation such a characterization was not possible, we conclude, based on the size of the dendritic area, that the layer 9 neurons will most likely have small RFs and respond to stationary stimuli with a high contrast.

Radial cell types in the avian TeO have been shown to project to specific nuclei such as the Glv or the isthmic nuclei. However, in the radial cells of layer 9, axons could not always be identified after intracellular filling. We nevertheless consider it unlikely that layer 9 neurons are without axons as some tectal cell types are, e.g., the horizontal cells in layer 5b (Luksch and Golz, 2003), and rather think that we missed axonal structures during reconstruction.

In respect to the neurotransmitter involved, layer 9 has been shown to be largely free of GABAergic and choline acetyltransferase-positive cells in previous immunohistochemical studies (Sorenson et al., 1989; Medina and Reiner, 1994; Tömböl, 1998). In other studies glutamate-, nitric oxide- and substance P-positive cells have been localized in layer 9, leaving those three as potential candidates (Morino et al., 1991; Bagnoli et al., 1992; Meyer et al., 1994). Thus, tectal input to the PL is very likely excitatory.

### Functional Considerations

The avian OT contains exquisite maps of space, both in respect to the visual modality and (at least in some species) the auditory modality. The dendrites of radial neurons usually sample only a small angle of that map, thus retaining a high resolution. Connections of such radial tectal neurons appear to be essential for the functions carried out by the isthmic system (Lai et al., 2011; Mysore and Knudsen, 2011), the isthmo-optic circuitry (Ohno and Uchiyama, 2009), and the connection to the thalamus (Vega-Zuniga et al., 2014). On the other hand, several output streams of the avian OT discard the high-resolution of the radial cell system. This is most prominent in the projection towards the nucleus rotundus, where retinotopy is discarded and a functional topography is established through the projection of the wide-field neurons of layer 13 (Wang et al., 1993; Marín et al., 2003). A second, inhibitory projection towards the nucleus rotundus *via* the nucleus subpretectalis also receives input from layer 13 (Theiss et al., 2003), leaving the major ascending tectofugal pathway without high spatial resolution.

Thus, the second ascending tectofugal pathway in birds is interesting as it receives wide-field input *via* layer 13 neurons, as well as an additional projection *via* the lateral pontine area, which originates from the layer 9 neurons in the tectum—which are radial, small-field neurons. Currently, one can only speculate that most of the projection is in fact directed at cerebellar circuits as proposed in pigeons (Clarke, 1977) and zebra finches (Wild and Gaede, 2016). In a study on the tectofugal projections of the pigeon, Hellmann et al. (2004) put forward the idea that the tectopontine pathway might be essential for avoidance behavior. This idea was taken up and discussed as a circuit for obstacle avoidance during flight in cluttered environments (Pakan and Wylie, 2006; Wylie et al., 2018), where it was argued that the tectopontine projection contributes local motion information. While this appears to hold for the projection to the pretectal nucleus lentiformis mesencephalic and the cerebellum, it is likely that spatial information will be discarded in the projection of the PL to the DLP. What the task of this additional tectal projection to the accessory tectofugal pathway is, thus remains enigmatic.

## Data Availability Statement

The raw data supporting the conclusions of this manuscript will be made available by the authors, without undue reservation, to any qualified researcher.

## Ethics Statement

The animal study was reviewed and approved by Regierung von Oberbayern, München, Germany, Permit 55.2-1.54-2532-32-11.

## Author Contributions

All authors had full access to all the data in the study and take responsibility for the integrity of the data and the accuracy of the data analysis. HL: study concept and design, study supervision. MK, SW and HL: acquisition of data and analysis and interpretation of data. MK and HL: drafting of the manuscript. HL and SW: critical revision of the manuscript for important intellectual content.

## Conflict of Interest

The authors declare that the research was conducted in the absence of any commercial or financial relationships that could be construed as a potential conflict of interest.
